# Serum protein electrophoresis patterns and misleading laboratory values in IgG4-RD: what the nephrologist should know

**DOI:** 10.1007/s40620-024-01992-x

**Published:** 2024-06-18

**Authors:** Eibhlin Goggins, William F. Glass, Corey Cavanaugh

**Affiliations:** 1 Division of Nephrology, University of Virginia School of Medicine, Charlottesville, VA USA; 2 Department of Pathology and Laboratory Medicine, University of Virginia Health System, Charlottesville, VA USA

**Keywords:** Immunoglobulin G4 (IgG4)-related disease, IgG4, Serum protein electrophoresis (SPEP), Nephritis, Kidney Biopsy

## Case presentation

A 66-year-old man with a recent history of stroke and chronic kidney disease (CKD) presented with unilateral weakness and falls. One month prior, he presented with diplopia, gait abnormalities, and hypertension and was found to have a pontine ischemic stroke and a creatinine of 1.8 mg/dL.

Upon current admission, he was found to have a creatinine of 4.8 mg/dL and a thalamocapsular infarct on non-contrast computed tomography (CT) scan. Additional workup revealed a protein gap on the metabolic panel, normocytic anemia, hyper-eosinophilia, lymphopenia, low C3 (67 mg/dL), mild liver function test elevation, and ultrasound imaging demonstrating increased echogenicity. Pertinent negative screens included rheumatoid factor (RF), glomerular basement membrane (GBM), and Anti-Neutrophil Cytoplasmic Antibody (ANCA) serology as well as HIV and HCV. Skeletal survey was normal. Medications included diltiazem and aspirin.

**Fig. 1:**
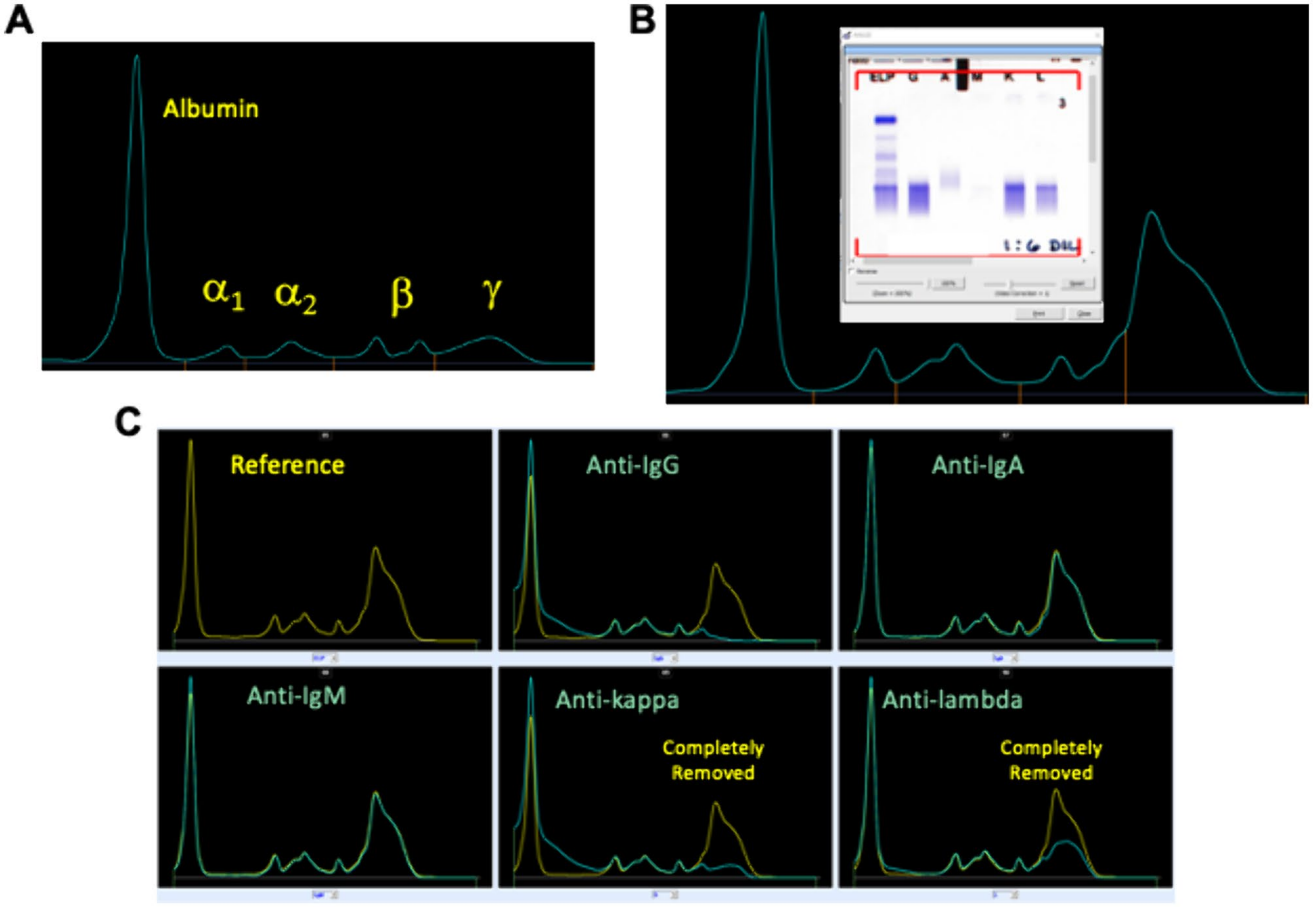
**A** Normal Serum Protein Electrophoresis Peak Pattern **B** Patient’s Serum Protein Electrophoresis Peak Pattern with Patient’s Serum Immunofixation Bands Pattern **C** Patient’s Serum Protein Electrophoresis after Immunosubtraction

Discordant urine protein and albumin were noted, with a urine protein/creatinine ratio of 1.15 g/g and a urine albumin/creatinine ratio of just 58 mg/g. Urine microscopy showed muddy brown casts and few renal tubular epithelial cells with no dysmorphic red blood cells or cellular casts. Serum protein electrophoresis revealed an abnormal band in the gamma region identified as IgG (Fig. 1). However, due to high heavy polyclonal background, the band could not be quantified. The band was immunotyped as both kappa and lambda by immunodeletion and immunosubtraction, raising the question of a dual monoclonal gammopathy. However, there was an elevated kappa/lambda ratio at 4.74 (106.27/22.40 mg/dL).

**Fig. 2:**
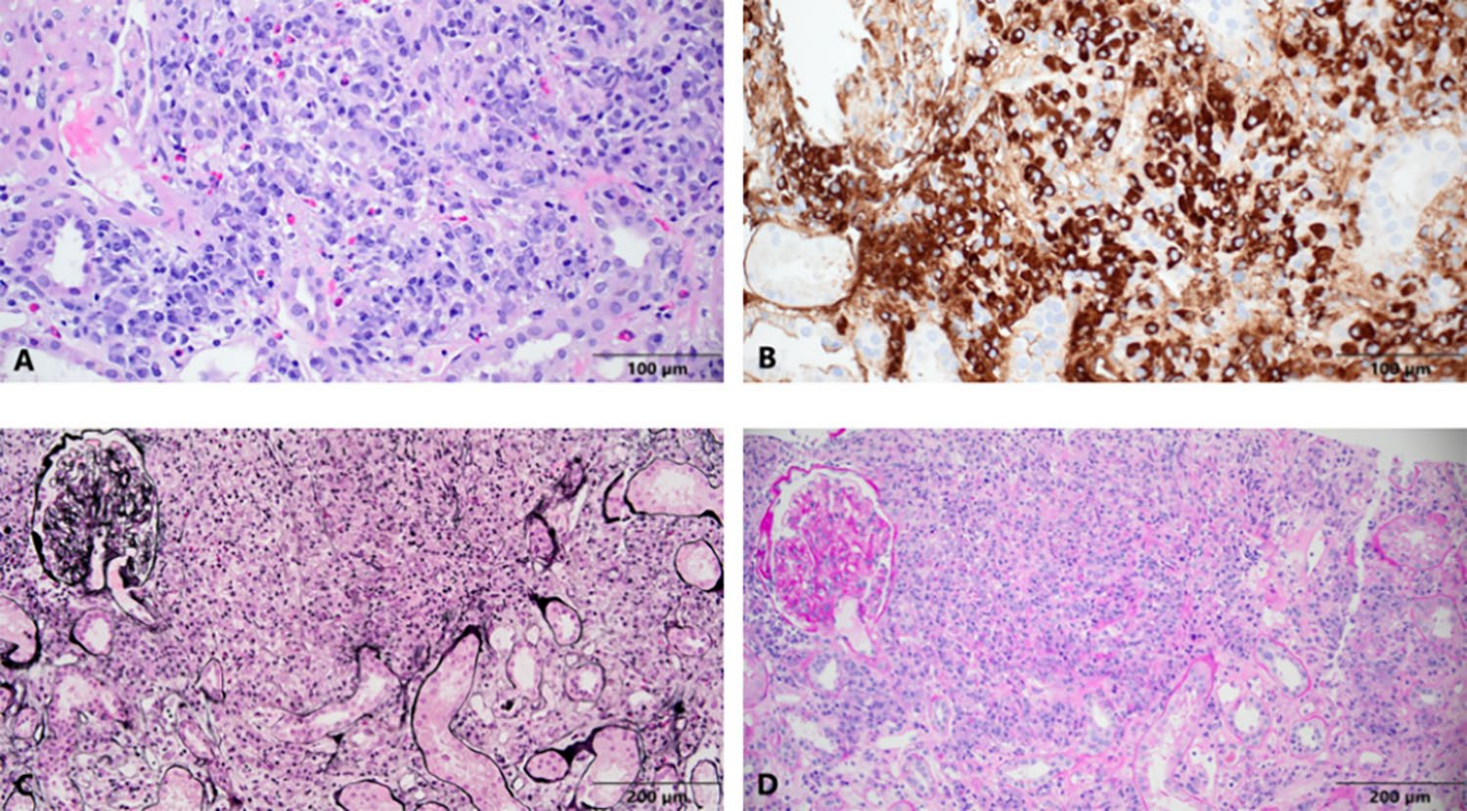
**A** Diffuse tubulointerstitial inflammation with abundant plasma cells, lymphocytes, and scattered eosinophils (H&E: 40 × objective). **B** Same field on adjacent slide with numerous (> 10 per high power field, HPF) IgG4-positive plasma cells (40 × objective). **C** Jones silver stain of same field showing focal tubular basement membrane disruption and tubular dropout. **D** Lower magnification showing diffuse plasma cell infiltrate and tubular dropout (Periodic Acid-Schiff Stain, PAS: 20 × objective)

Bone marrow biopsy showed normocellular bone marrow with trilineage hematopoiesis, ringed sideroblasts (~ 10%), and a mild increase in polytypic plasma cells. However, there was no evidence of malignancy, B-cell or plasma cell clone. A renal biopsy was obtained and was notable for severe active and early chronic tubulointerstitial nephritis with abundant plasma cells and lymphocytes, as well as widely scattered eosinophils and some focally clustered eosinophils **(**Fig. 2**)**. There was extensive (50–60%) interstitial edema and tubular dropout. Although storiform and bird's eye fibrosis were absent, the possibility of IgG4-related disease was considered due to the abundance of plasma cells. Immunohistochemistry for IgG4 and IgG were performed, revealing that the number of IgG4-positive plasma cells greatly exceeded 40% of the plasma cell population with focal areas having over 200 IgG4-positive plasma cells per high power field **(**Fig. 2B**)**. However, IgG subclassing unexpectedly showed normal IgG4 (48.2 mg/dL). Immunofluorescence done at the time of biopsy was noncontributory.

The patient’s samples were sent to the Mayo Clinic for further testing. Urine protein electrophoresis demonstrated free kappa light chain at the beta-gamma order. Additionally, clinical suspicion for IgG4-Related Disease (IgG4-RD) prompted a repeat of IgG subtyping with sample dilution. Corrected lab results revealed an extremely elevated IgG4 of 1540 mg/dL **(**Table 1**)**. These new results, coupled with the kidney biopsy findings, strongly favored IgG4-related disease.

**Table 1:** Patient’s Immunoglobulin, IgG4 subclass (After Correction), and Serum Free Light Chain Values

	Ref range & units	Patient’s values
IgA	767–1590 mg/dL	179.0
IgM	22.0–240.0 mg/dL	69.0
Total IgG subclasses	767–1590 mg/dL	3030
IgG1	341–894 mg/dL	2050
IgG2	171–632 mg/dL	554
IgG3	18.4–106.0 mg/dL	236.0
IgG4	2.4–121.0 mg/dL	1540.0
Serum free kappa light chain	0.33–1.94 mg/dL	106.27
Serum free lambda light chain	0.57–2.63 mg/dL	22.40
Kappa/Lambda FLC ratio	0.26–1.65 ratio	4.74

## Major teaching points

- IgG4-RD is a systemic immune-mediated fibroinflammatory condition with a wide variability in clinical presentation and laboratory findings.
- IgG4 can have a unique migration pattern on serum protein electrophoresis resulting in a narrow band that may mimic a monoclonal gammopathy.
- Elevated serum IgG4 is not specific for IgG4-RD. Furthermore, because IgG4 subclass reagents are designed to measure subclass deficiencies rather than excesses, additional dilutions may be needed to identify an excess of IgG4.
- An appreciation for the varied presentation of IgG4-RD is crucial for improving patient care and for undertaking further research related to the disease.

## Lessons for the clinical nephrologist

Immunoglobulin G4-related disease is a systemic immune-mediated fibroinflammatory condition affecting numerous organs. It is characterized by an increase in serum IgG4 levels and an infiltration of IgG4-positive cells into multiple organs. IgG4-RD was first described as a unique clinical entity in 2003 [1]. Since then, our understanding has grown substantially, however there is still much to learn regarding the pathogenesis, diagnosis, and treatment. This case illustrates one of the many clinical presentations of IgG4-RD and highlights that routine diagnostic workup may be insufficient to identify the disease.

In clinical practice, diagnosis of IgG4-RD is typically made based on elevated serum IgG4 and an infiltration of IgG4-positive cells into multiple organs. However, determination of the most appropriate diagnostic criteria for IgG4-RD has been a challenge due to its wide variability in presentation. Recently, a revised comprehensive diagnostic criterion for IgG4-RD was released by a working group that included physicians from multiple specialties [2]. Three domains for diagnosis were included: clinical and radiological features, serological diagnosis and pathological diagnosis [2].

The most commonly involved organs in IgG4-RD include the pancreas, biliary duct, kidneys, lungs, thyroid, and salivary glands, although involvement of every organ system has been reported. Furthermore, the condition can have varying effects within each organ. The kidneys may be involved in a quarter of cases [3], but can present as tubulointerstitial nephritis, membranous glomerulonephritis or pyelitis. There may be an insidious or rapidly progressive rise in creatinine with or without proteinuria. Because the clinical manifestations of IgG4-RD are widespread, it can mimic several autoimmune, malignant, and rheumatologic diseases. Indeed, there have been multiple reports of IgG4-RD patients that were initially misdiagnosed with malignant neoplasms, lymphomas, and various other diseases such as Sjögren’s syndrome [4, 5]. In the case of our patient, despite consultations with numerous clinicians, prior to outside testing, the differential diagnosis remained long and included light chain cast nephropathy, paraprotein-related kidney disease, IgG4-RD and C3 GN (with low C3). Had IgG4-RD not remained on the differential, our patient may not have received optimal clinical management.

The variability in presentation is further complicated by variability in laboratory findings. IgG4 has unique functional and structural properties and, unlike other IgG subtypes, IgG4 cannot fix complement [4]. Serum protein electrophoresis is a test commonly included in the work up of patients presenting with the constellation of symptoms seen in IgG4-RD. However, the migration pattern of IgG4 on serum protein electrophoresis can be misleading and may be misinterpreted as a biclonal IgG kappa and lambda gammopathy, a polyclonal increase in IgA, or a monoclonal gammopathy. IgG4 may form a narrow band in the anodal γ region on serum protein electrophoresis, a pattern resembling monoclonal gammopathies [6]. This is complicated in a subset of patients with apparent kappa restriction [6]. Additionally, it has been documented that IgG4 molecules can undergo Fab arm swapping due to loose binding, to yield a bispecific Ig containing both kappa and lambda, giving the impression of a dual monoclonal gammopathy. The instability of the disulfide bonds between IgG4 heavy chains can result in a single light chain/single heavy chain combination [7]. Thus, serum protein electrophoresis should be carefully interpreted alongside other findings and is insufficient on its own to identify the disease.

Clinicians often rely heavily on serum IgG4 to diagnose or rule out IgG4-RD. However, serum IgG4 levels can vary widely and normal IgG4 levels have been reported in patients with biopsy-proven disease. Additionally, elevated IgG4 is not specific to IgG4-RD and can be seen in a variety of diseases such as systemic vasculitis, respiratory diseases, and multicentric Castleman’s disease [5]. Furthermore, IgG4 assays are susceptible to the prozone or ‘hook’ effect, which occurs when excess antigen interferes with immune complex formation, leading to an underestimation of the antigen being measured [5, 8]. This can be easily overcome by performing sample dilutions: a higher value obtained from the diluted sample compared to the undiluted sample suggests that the prozone effect is present. However, unless there is a high degree of clinical suspicion for IgG4-RD, this may not be undertaken. The prozone effect should be considered when there is discrepancy between serum IgG4 levels and clinical findings suggesting IgG4-RD. This has led to diagnostic and treatment delays and has likely contributed to an underreporting of the disease. In our case, although renal biopsy findings suggested the possibility of IgG4-RD, original IgG subclass testing erroneously showed normal IgG4 levels. Our suspicion for IgG4-RD prompted us to continue pursuing this possibility and a highly elevated IgG4 level was revealed only after performing serial dilutions. Thus, in order to reach the final diagnosis in this patient, it was necessary to go beyond routine diagnostic workup and consider all findings, including clinical, pathological and laboratory results, together.

First-line induction therapy for IgG4-RD includes corticosteroids and patients often have a rapid and robust response [9]. Therefore, proper and timely identification of the disease is crucial. Once our patient was properly diagnosed, he was treated with, and responded well to, prednisone (creatinine decreased to 1.5 mg/dL). For patients who do not improve with steroids or experience a relapse, steroid-sparing agents, such as rituximab, may be considered. Additional options like azathioprine, mycophenolate mofetil, methotrexate, and tacrolimus have also been suggested [9]. Trials assessing the efficacy of targeted therapies for patients with or at risk of complications are currently underway, however, there are no approved therapies for IgG4-RD.

In conclusion, IgG4-RD is a unique disease with a wide variability in clinical presentation and laboratory findings. The diagnosis often requires multiple findings to be interpreted together. We therefore encourage clinicians to consider IgG4-RD even when all the findings do not seem to fit together as expected.

## Data Availability

N/A.

## Abbreviations

ANCA: Anti-neutrophil cytoplasmic antibody
CKD: Chronic kidney disease
GBM: Glomerular basement membrane
IgG4-RD: Immunoglobulin G4-related disease
LFT: Liver function test
RF: Rheumatoid factor
SPEP: Serum protein electrophoresis
UPEP: Urine protein electrophoresis
